# Reusable Turn-on Fluorescent Biosensor for Cardiac Biomarker Troponin I Detection Using QDs-SPION-Aptamer

**DOI:** 10.1007/s10895-025-04303-0

**Published:** 2025-04-21

**Authors:** Ayse Ozdemir, Yigitcan Algül, Nimet Yildirim Tirgil

**Affiliations:** 1 Software Engineering, Ankara Bilim University, Ankara, Turkey; 2 Institute of Graduate Programs and Department of Biology, Polatlı Science and Literature Faculty, Ankara Hacı Bayram Veli University, 06900 Ankara, Turkey; 3 Nanosan Laboratory, Department of Biology, Polatlı Science and Literature Faculty, Ankara Hacı Bayram Veli University, 06900 Ankara, Turkey; 4 Biomedical Engineering Department, Faculty of Engineering and Natural Sciences, Ankara Yildirim Beyazıt University, Ankara, Turkey; 5 Metallurgical and Materials Engineering Department, Faculty of Engineering and Natural Sciences, Ankara Yıldırım Beyazıt University, Ankara, Turkey

**Keywords:** Quantum dots, Superparamagnetic iron oxide nanoparticles, Aptamer, Fluorescence

## Abstract

Tro ponin is a biomolecule found in the blood of humans that can be used as an indicator of cardiac damage and cardiovascular risk. In this study, a turn-on fluorescence-based biosensor was developed using CdSe/ZnS quantum nanoparticles with high fluorescence efficiency decorated on superparamagnetic nanoparticles and aptamer as a probe for cardiac troponin I detection to improve fluorescence efficiency and create a reusable sensor through magnetic separation. Chemical modifications of biosensor components have been characterized by zeta sizer, zeta potential, UV–Vis spectroscopy, transmission electron microscopy (TEM), and Fourier transform infrared spectroscopy (FTIR). We reported that the developed sensor worked with a "Turn-on" mechanism. Fluorescence changes of the developed aptamer-based biosensor were evaluated using a fluorometer, and a low limit of detection (LOD) of 13.3 ng/ml with a linear detection range from 2 to 40 ng/ml was determined. Additionally, the selectivity and sensitivity of the biosensor have been tested with several biomarkers found in human blood.

**Graphical Abstract:**

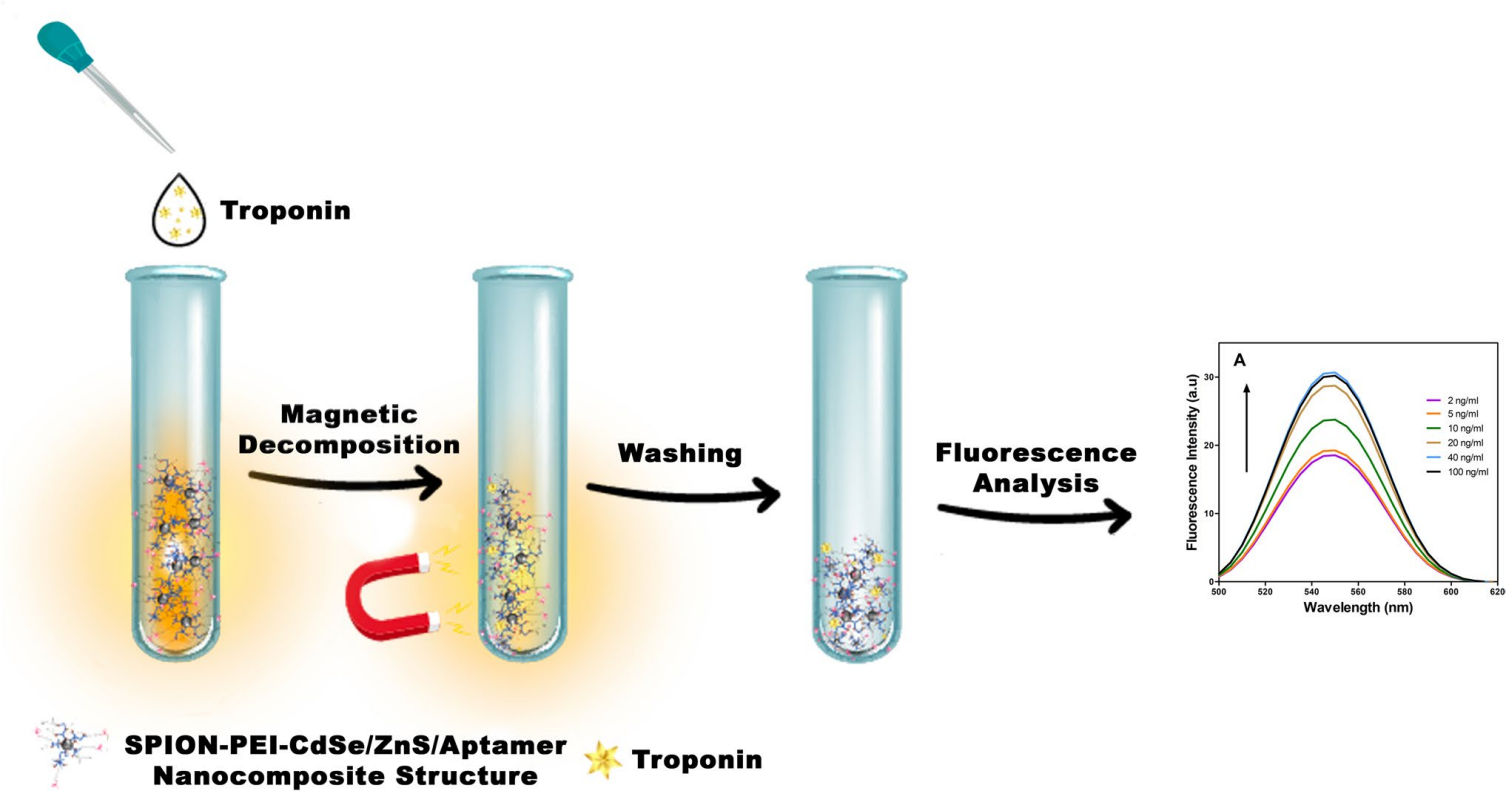

**Supplementary Information:**

The online version contains supplementary material available at 10.1007/s10895-025-04303-0.

## Introduction

Biosensor technologies are a field of study that requires linking biotechnology and nanotechnology with a multi-disciplinary approach. With the help of biosensors, it is possible to identify many different biological molecules, foodborne pathogens, analysis of physical, chemical or radiological contaminants in water is possible [1, 2].Compared to traditional methods, biosensors have the advantages of being easy, fast and stable. The best known and widely used biosensors (also known as Point-of-Care Testing) are amperometric or potentiometric enzyme-based systems for the determination of glucose from biological samples (blood, saliva, etc.). The enzyme glucose oxidase immobilized on the biosensor reacts in the presence of glucose to produce hydrogen peroxide. This can be measured amperometrically at the anode. Many companies such as Bayer®, Nova Biyomedical®, Roche®, LifeScan® have commercialized various biosensors that can measure glucose levels in the range of 10 - 600 mg/dL from 0.3 - 1 μl sample.

Nano-biosensors are a new field that started to develop after 1990 with the development of technology. Nanobiosensors are analytical devices that combine a biologically sensitive element with a nanostructured transducer and have attracted considerable attention in the contemporary scientific community. These sensors are measuring devices comprising nano-sized components that are capable of analysing a range of biological molecules present in a minimal volume of bodily fluid, such as blood or saliva. Nanobiosensors can perform ultra-sensitive measurements and can detect extremely low levels of analytes, including picomoles per liter. Nanobiosensors offer several advantages over traditional laboratory methods due to their inherent specificity, simplicity and rapid response. In particular, they are proving invaluable for the early diagnosis of diseases through the use of disease markers, with optical, physical and electrochemical nanobiosensors becoming increasingly prevalent as they are integrated with smart mobile devices [3]. It is anticipated that nanobiosensors will become a key diagnostic tool in the future, offering sensitivity, specificity and reliability in medical applications. Nanobiosensors have different names depending on the material used, how it is analyzed and even the biological molecules analyzed. Optical biosensors represent the most varied group of biosensors, encompassing different types of spectroscopies, including fluorescence, phosphorescence, or absorption. Optical sensors based on fluorescence measurement can be decorated with various molecules to provide a unique binding to the target molecule [4].

Aptamers are short, synthetic DNA or RNA molecules, consisting of approximately 12–80 nucleotides, that have been engineered to specifically bind to a chosen target biological molecule. These aptamers are selected through a process known as SELEX (Systematic Evolution of Ligands by Exponential Enrichment), where they are isolated from a pool of sequences after multiple rounds of selection. This process typically yields multiple nucleotide sequences with varying affinities for the target molecule. The target molecule is then assessed with a dissociation constant (KD), and the aptamer sequence with the lowest KD (highest affinity) is chosen as the aptamer of interest. Aptamers offer several advantages over other binding molecules like antibodies and enzymes, including increased stability, ease of storage, synthetic production, and resilience to environmental conditions and flexibility [5, 6].

Aptamer-based biosensors that utilize ligand binding can be developed to detect a wide range of analytes in various ways. The most common analytical method involves monitoring changes in optical signals due to fluorescence alterations [7]. One of the earliest examples of an optical aptasensor was a thrombin-specific binding aptasensor labeled with a fluorophore and immobilized on a glass surface [7, 8]. One of the earliest examples of an optical aptasensor was a thrombin-specific binding aptasensor labeled with a fluorophore and immobilized on a glass surface. This biosensor demonstrated the ability to detect 5 nM thrombin with less than 4% error. Additionally, nanoparticles have been incorporated into biosensor designs to enhance the binding affinity of the aptamer to the ligand and improve the optical signal. In a study involving anti-cancer cell aptamers, aptamers labeled with 80 different fluorophores were attached to nanorods and subsequently analyzed. The results showed a 26-fold increase in affinity and a 300-fold increase in fluorescence intensity [9].

The American College of Cardiology (ACC) and the European Society of Cardiology (ESC) have established criteria for diagnosing a heart attack (acute myocardial infarction - AMI). In the event of a heart attack, it is anticipated that the levels of serum troponins (TnT or TnI) or cardiac biomarkers will deviate from normal values

[10]. The American College of Cardiology (ACC) and the European Society of Cardiology (ESC) have established criteria for diagnosing a heart attack (acute myocardial infarction - AMI). In the event of a heart attack, it is anticipated that the levels of serum troponins (TnT or TnI) or cardiac biomarkers will deviate from normal value [11]. Troponin is a highly complex molecule composed of three regulatory proteins called troponin C, troponin I, and troponin T. A heart attack caused by insufficient nourishment of the heart tissue or blockage of coronary arteries can be detected by examining troponin I and troponin T levels in the serum. It is known that in the case of a heart attack or ischemia, troponin levels reach a maximum within 1–2 days and continue to be present in the blood in significant amounts even after 10 days [12]. There are several studies aimed at understanding the long-term effects of Troponin I. The lowest detection limit of cardiac troponin I in serum is 0.006 ng/mL [13]. The standard threshold point is determined according to the 99 percent rule, and values greater than 0.04 ng/mL are defined as troponin elevation. The reference value for troponin in blood in routine tests to assess the risk of a heart attack in our country is 0–16 ng/mL. In a study comparing the results of the POC method used in troponin I analysis and the laboratory test (i-STAT cardiac troponin I test, Abbott Point of Care) in patients with suspected acute coronary syndrome, blood samples were analyzed simultaneously. When the results were analyzed, no statistical difference was found [14]. The quantitatively detectable lowest and highest values (LOD) vary depending on the analysis method used and the sensitivity of the sensor [15]. Laboratory tests require skilled personnel and high-cost devices. However, Troponin analysis with a nanobiosensor has the advantages of being cheap and easy.

Superparamagnetic nanoparticles have become preferred materials in biomedical applications, especially in biosensor designs, due to their extremely small size and unique properties. Superparamagnetic nanoparticles have high saturation magnetization and initial permeability magnetization in the hysteresis loop [16]. These nanoparticles can be decorated with antibodies, aptamers, ligands, or imaging agents to form a drug delivery system, imaging system, or sensor system. Additionally, these modifications reduce the cytotoxicity of the nanoparticles [17]. In this project, iron oxide nanoparticles functionalized with biocompatible polyethylene glycol were designed to reduce toxicity and enable the binding of other active groups.

Quantum dots (QDs) are interesting and useful nanotechnology materials. They were discovered by Alexie Ekimov in the 1980’s. They are artificial sub- 100 nm semiconductor nanoparticles with excitons confined in all three dimensions, such as carbon, silver, gold and silicon quantum dots, etc. Drug delivery, bioimaging and sensing are possible applications for quantum dot [18]. Quantum nanoparticles are semiconductor nanocrystalline structures that can fluoresce at certain wavelengths [19]. They are usually composed of between one hundred and one hundred thousand atoms and have dimensions below 100 nm. Their electrical energy levels and extremely small size enable these materials to exhibit characteristic optical properties. In recent years, the creation of water-soluble quantum nanoparticle structures and various biomolecules (proteins, carbohydrates, aptamers, etc.) binding, they have been frequently used in biomolecular labeling and cell imaging [20]. Organic dyes and proteinaceous materials that fluoresce when excited with light have various disadvantages such as low fluorescence lifetime and fluorescence efficiency, spontaneous quenching, pH-dependent changes, and stability problems in aqueous solutions. On the other hand, quantum nanoparticles have size-dependent tunable light emission, resistance to light bleaching, and simultaneous excitation of fluorescent material at different wavelengths have made them an interesting material in biosensor designs [21].

One of the most widely used QDs in nanobiosensor designs is CdSe/ZnS. ZnS coating is used to cover the defects on the CdSe shell surface and to increase the luminescence quantum yield of the material. The quantum efficiency of bifunctional (magnetic and fluorescent) Fe3O4/CdSe/ZnS nanocomposites increased by 2–3% to 10–15% compared to before the ZnS layer was coated [22]. The binding of biomolecules (protein, antibody, aptamer, etc.) to QDs is generally based on electrostatic interactions. This binding creates a bioluminescent signal, and the changes that occur can be analyzed by spectroscopic methods.

The alteration in photoluminescence value, spectral shifts, full width at half maximum (FWHM), and the full width at half height provide information about the state of the system before and following binding with the biomolecule. It is established in the literature that aptamers can be readily adsorbed onto the surface of QDs [23].

In this work, we developed a novel and easy platform for detecting a cardiac biomarker. This study aims to produce and test a nanocomposite biosensor to diagnose troponin, a biomolecule found in the blood in case of a heart attack (acute myocardial infarction—AMI). We developed a system based on the binding of quantum crystals to the target analyte, which changes their optical properties. The objective of this study is to develop a nanobiosensor for the analysis of Troponin I with a sensitivity of pg/ml. The sensor will be decorated with an aptamer that enables specific binding to fluorescent QDs, which will be purified by separation with nanoparticles. A comparison of the performance of different sensing methods for troponin is summarized in Table 1. A literature review reveals a considerable range in the reported detection limits of troponin sensors, with values ranging from 250 ng/ml to 0.1 pg/ml. This variability is attributed to the differing methodologies employed in the various studies.

**Table 1:** Overview for different sensing systems for Troponin I detection

Sensing system	probe	LOD	References
Integrated Electrode	aptamer	24 pg/mL	[12]
Integrated Electrode	aptamer- gold	2.4 pg/mL	[12]
Surface plasmon resonance	monoclonal antibody	250 ng/mL	[24]
Chemi- luminesence	antibody	27 ng/mL	[25]
Colorimetry	monoclonal antibody	10 ng/mL	[26]
Silicon-FET	monoclonal antibody	92 pg/mL	[27]
Graphene-FET	monoclonal antibody	0.1 pg/mL	[28]
Zinc oxide- FET	monoclonal antibody	3.24 pg/mL	[29]
Fluorescence	CdSe/ZnS-SPION-Aptamer sensor	13.3 ng/ml	This work

Compared to other sensor designs for cardiac biomarkers, the CdSe/ZnS-SPION-Aptamer biosensing probe for troponin offers improved stability in biological environments and reusability with magnetic separation without significant change in sensor fluorescence. In addition, aptamer-based sensors are more cost-effective and suitable for large-scale production than conventional monoclonal antibody-integrated sensors [30].

## Material and Methods

### Reagents and Chemicals

2-N-morpholinoethanesulfonic acid (MES) (pH 5.8) from BioShop (Canada, Burlington), 3-mercaptopropionic acid (MPA), 3-ethylcarbodiimide hydrochloride (EDC), N-hydroxysuccinimide (NHS), uric acid, lactate, glucose, Human Serum Albumin (HSA) were supplied from Sigma-Aldrich (Shanghai, China).

CdSe/ZnS Q-Dots were kindly donated by Kelestemur group which prepared by the modified Bae’s protocol [31]. The Mavi Group has kindly made a donation of polyethylenimine (PEI) coated water soluble SPIONs, which have been prepared using the methodology outlined in this article [32]. The Tro4 aptamer with sequence 5’CGTGCAGTACGCCAACCTTTCTCATGCGCTGCCCCTCTTA3’ modified with C6 spacer was purchased from Motex. Aliquots of troponin were prepared in enzyme-free double-distilled water. Deionized water (resistivity 18.2 Mcm^−1^) was used to prepare chemical reagents without further modifications.

### Methods and Protocols

#### Preparation of QDs-SPION-Aptamer

To facilitate the biosensor’s functioning in an aqueous environment, water-soluble materials were obtained through the coating of CdSe/ZnS QDs with 3-mercaptopropanoic acid (MPA), which contains carboxylic acid and thiol groups and has functional groups that render the surface on which they are coated negatively charged. The carboxyl groups were activated using N-(3-dimethylaminopropyl)-N-ethylcarbodiimide (EDC) and N-hydroxysuccinimide (NHS). The Tro4 aptamer was attached to the activated SPION/QD conjugates.

In this work, CdSe/ZnS QDs in organic solvent have been transferred to the water. MPA was used to functionalize the carboxyl (-COOH) groups on the CdSe/ZnS Q-Dots. 50 μg/mL SPION (14.3 μg/mL), 5 mg/mL Q-dot, 120 mg/mL EDC and MES buffer were added to a test tube and sonicated for 30 min. Then 120 mg/mL NHS was added and sonicated for 60 min. After sonication, the sensor solution was divided into 3 separate test tubes, then Tro- 4 aptamer was added (40 μg/ml) and incubated in a rotator shaker for 1 h.

#### Characterization

Morphological evaluation was performed by transmission electron microscopy (TEM, FEI Tecnai G2 F30). 10 μL of 50-fold diluted samples were applied to a copper grid coated with carbon film (300 mesh). The samples were incubated for 10 min. Pipette off excess solution and allow to dry for 24 h at room temperature. Fourier Infrared spectra (FT-IR, FT/IR- 6X FTIR spectrophotometer) were obtained using an infrared spectrometer.

Dynamic Light Scattering (DLS) was used to measure the hydrodynamic sizes and zeta potentials of the nanobiosensor and its components. The DLS was performed using the Malvern Nanosizer/Zetasizer ZEN 3600 (Malvern Instruments, USA) with 173° detector angle. Measurements were made in quartz cuvettes for hydrophilic SPION, QDs, SPION + QDs, and nanocomposite biosensor diluted in double-distilled water (ddH_2_O). Zeta size measurements had 11 runs within 10 s. Dipping cell electrodes were used to measure zeta potential. The refractive index, viscosity, and dielectric constant values for experiments performed in ddH_2_O at 25 °C were 1.33, 0.8872, and 78.5, respectively. The concentration of QDs, SPION was set at 0.5, 0.01 mg mL^−1^ for all DLS experiments.

The optical properties of the developed nanosensor were analyzed by UV–Visible spectroscopy. The UV − vis measurements were accomplished with a Denovix® UV − vis spectrophotometer using quartz cuvettes with an optical path length of 10 mm.

#### Fluorescence Spectroscopy Analysis

Fluorescence spectroscopy was employed to assess the binding interactions between the QDs-SPION-Aptamer conjugates and the target molecule (Tro). Fluorescence spectra were recorded using a QFX Fluorometer (Denovix) and the measurements were conducted over a wavelength range of 500 nm to 700 nm using a Varioskan Flash Multimode Spectrophotometer (Thermo Fisher Scientific).

For each measurement, the fluorescence emission spectra were obtained with an excitation wavelength set at 430 nm. The emission spectra were recorded within the 500–600 nm range. To ensure the accuracy and reliability of the fluorescence measurements, the experiments were performed using two different optical filters: a blue filter and a UV filter. Each set of measurements was conducted separately under identical conditions to facilitate a direct comparison of the fluorescence response under different filtering conditions.

The resulting fluorescence data were used to evaluate the binding efficiency and specificity of the QDs-SPION-Aptamer-based biosensor system, providing critical insights into the performance of the biosensor under varying experimental conditions. In order to estimate the lower LOD, a formula was employed: LOD = 3SD/m, where SD represents the standard deviation of repeated measurements of the standard solution at the lowest concentration, and m denotes the slope of the calibration curve [33].

#### Stability and Selectivity of QDs-SPION-Aptamer Biosensor

Fluorescence spectroscopy was utilized to evaluate the selectivity of the biosensor towards various analytes, including ascorbic acid, uric acid, lactate, glucose, human serum albumin (HSA), hypoxanthine, inosine, phenol, dopamine, and Troponin, each at a concentration of 0.5 μg/mL. The fluorescence measurements were performed using a QFX Fluorometer (Denovix) over a wavelength range of 500 nm to 700 nm. For the before experiments, only the QDs-SPION-Aptamer nanobiosensor and Tris-HCl buffer were added to individual tubes labeled with the name of the specific analyte. For the after experiments, the analyte was dropped on the tubes and rotated for 30 minutes at room temperature. The final volume in each well was adjusted to ensure consistent conditions across all experiments. All the experimental groups normalized to the free sensor fluorescence value for the evaluation.

The stability and reusability of the developed QDs-SPION-Aptamer biosensor system were evaluated using Troponin as the target analyte after being used for other experiments and magnetically separated. Troponin was prepared at a stock concentration of 0.5 μg/mL. For the stability tests, Troponin was initially diluted to a concentration of 40 and 10 ng/mL. Three separate test tubes were prepared, each containing the QDs-SPION-Aptamer system and Troponin at two different concentrations. The samples were subjected to sonication for 30 minutes to ensure uniform dispersion and interaction between the biosensor components and the analyte. After sonication, the fluorescence of each sample was measured. Following the initial fluorescence measurement, additional Troponin was added to each test tube to achieve a final 40 ng/mL concentration. Fluorescence was then re-quantified to assess any changes in the biosensor’s performance and to determine the stability of the QDs-SPION-Aptamer system under increasing analyte concentrations. This procedure allowed for evaluating the biosensor’s stability and its ability to maintain consistent fluorescence signals in the presence of varying Troponin concentrations within a month, which is critical for its application in diagnostic assays.

#### Statistical Analysis

All analyses were performed in triplicate, and standard deviations were calculated from the mean of the data (SEM). Graphs were generated with GraphPad Prism 5 software® (La Jolla, CA). One-way analysis of variance (ANOVA) with Tukey’s test was used to evaluate group comparisons. p-values less than 0.05 were considered statistically significant.

## Results and Discussion

### Characterization of Prepared QDs-SPION-Aptamer Biosensor

In the design of the nanobiosensor, CdSe/ZnS QDs were utilized due to their common use in biosensor applications. The ZnS coating on the CdSe core was specifically selected to reduce surface defects and improve the fluorescent quantum yield of QDs, thereby enhancing their performance in fluorescence-based assays. Alongside QDs, superparamagnetic iron oxide nanoparticles (SPIONs) coated with PEI, a polymer rich in amine groups, were incorporated into the sensor design to enhance the nanosensor’s biocompatibility and reusability. The amine groups on the PEI coating facilitated the attachment of aptamers to the SPIONs, creating a robust and functional biosensor platform.

**Fig. 1:**
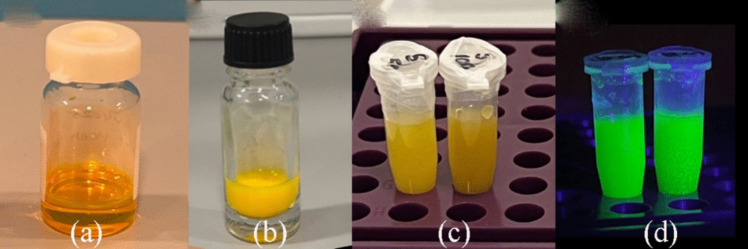
(**a**) Quantum crystals were obtained following a repeated transfer to water protocol, where the temperature and (**b**) MPA amount were adjusted, Examples of nanocomposite biosensors (**c**) under daylight, (**d**) under UV light

The aptamer loading on the nanobiosensor was quantified by measuring the absorption of single-stranded DNA (ssDNA) at 260 nm using a spectrophotometer. The initial concentration of aptamers was determined before conjugation, and the remaining amount on the sensor after the conjugation process was calculated. It was determined that approximately 70% of the initial aptamer content remained attached to the nanosensor, indicating effective functionalization of the QDs-SPION-Aptamer system (Sup.1). This retention rate is crucial for ensuring the biosensor’s specificity and sensitivity in detecting target analytes (Fig. 1).

The analysis of the hydrodynamic size of SPIONs, QDs, SPION + QD, and SPION + QD + aptamer (nanobiosensor) revealed sizes of 215.8 ± 3.22, 1929.33 ± 184, 2531.33 ± 211.3, and 5540.66 ± 2011 nm, respectively. The larger hydrodynamic size and standard deviation of QDs may be due to aggregate formation in an aqueous solution [34]. Our results indicate that the chemical binding of SPIONs, QDs, and aptamers leads to the formation of large colloidal particle sizes due to the shells, surface modifications, and electrostatic interactions [35].

Zeta potential (ξ potential) measurements of the components of the nanocomposite biosensor were subsequently analyzed to assess dynamic changes during sensor fabrication. The coating with MPA resulted in negatively charged carboxyl groups on the surface of CdSe/ZnS, creating a negative zeta potential around − 12 mV. While SPIONs exhibited an average + 15 mV ξ potential, there was a significant decrease in the zeta potential of SPIONs + QDs complexes to + 5 mV. The shift in zeta potential from positive values to − 32 mV indicated the successful aptamer coating of the nanosensor.

**Fig. 2:**
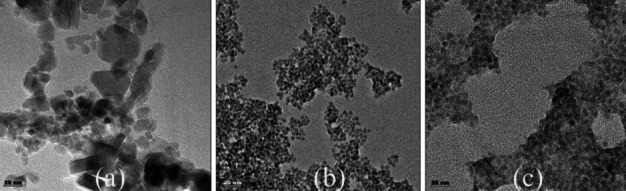
The TEM images of (**a**) SPIONs, (**b**) CdSe/ZnS QDs, and (**c**) CdSe/ZnS-SPION-Aptamer sensor, scale bar: 20 nm

Transmission electron microscopy (TEM) images showed that the core structures of MPA-coated QDs had a diameter of 10–15 nm and a circular morphology. While most PEI-coated SPIONs displayed a spherical morphology, some morphological changes, likely due to the coating, were observed. In contrast to the average hydrodynamic size of 215 nm as determined by zeta measurements, smaller particles were visible in TEM images. The PEI-coated SPIONs and CdSe/ZnS QDs were observed in their original, unaltered structures within the sensor. Additionally, TEM images indicated a high level of homogeneity and a spherical morphology in the nanocomposite structure. (Fig. 2)

**Fig. 3:**
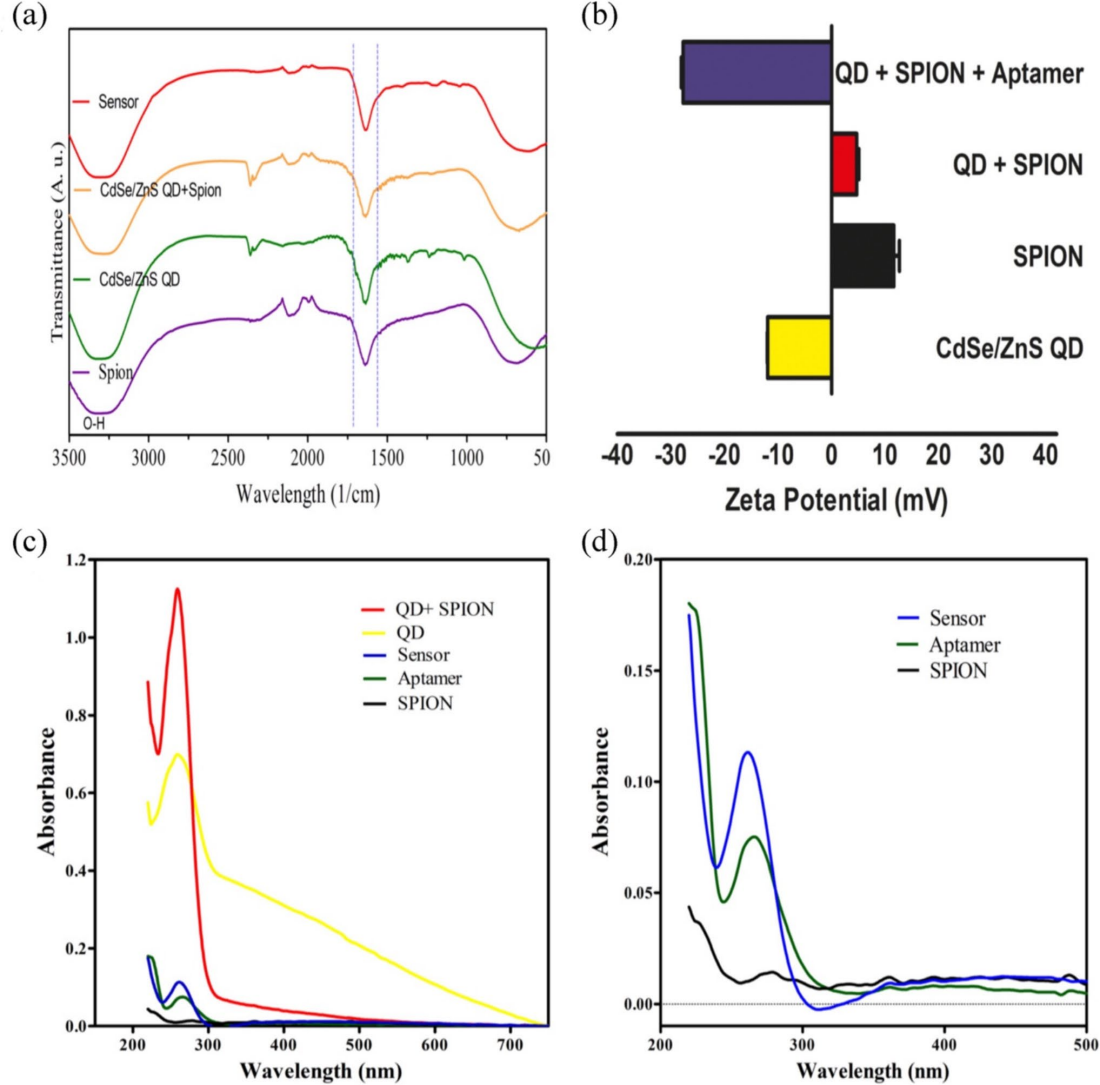
(**a**) FTIR spectra of SPIONs, QDs + SPIONs, and nanosensor, (**b**) The measured zeta potential of QDs, SPIONs, QDs + SPIONs, and QDs + SPIONs + aptamer conjugated nanosensor, (**c**) UV–Vis spectra of free components of the sensor separately, (**d**) absorbance at successive steps of fabrication

**Fig. 4:**
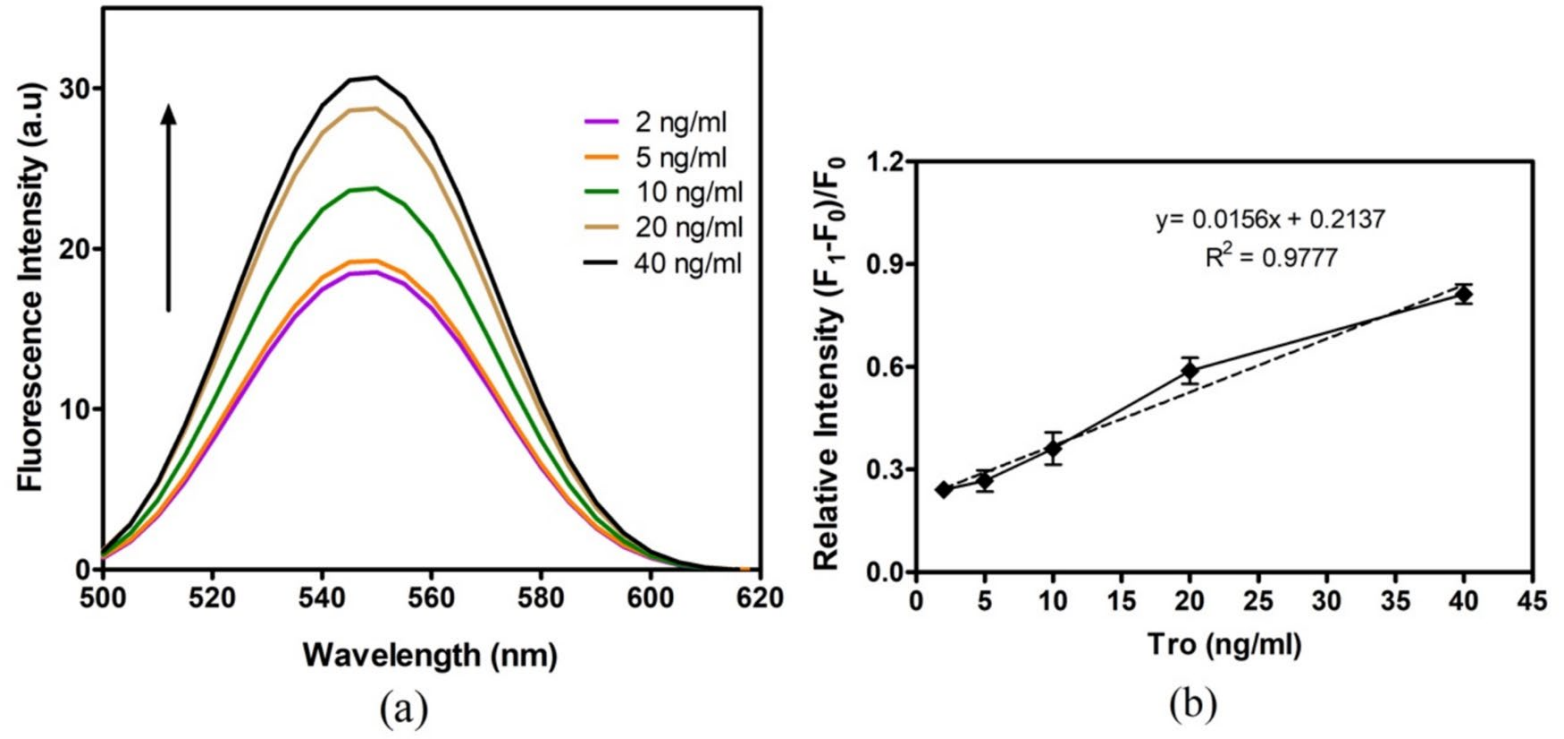
Determination of the analytical performance of CdSe/ZnS-SPION-Aptamer sensor (**a**) fluorescence spectra of nanobiosensor interaction with Troponin at different concentrations, (**b**) linear regression analysis showing relative fluorescence efficiency as a function of different troponin concentrations (S/N = 3)

Fourier-transform infrared spectroscopy (FTIR) is a widely used technique to identify changes in the vibrations of functional groups on a material’s surface. By comparing the frequency and location of these peaks before and after binding processes, the groups involved in binding or absorption can be analyzed [36]. FTIR spectra of SPION, QDs, SPION + QDs, and SPION + QDs + aptamer were obtained in the range of 3500–500 cm^−1^ (Fig. 3a). The vibration band related to the carboxyl in the MPA structure was observed at 500 cm^−1^. This carboxyl group played a role in surface binding, as evidenced by the disappearance of disulfide stretching in this range in QDs, making them water-soluble by exchanging the ligand on the surface with MPA. Additionally, the peak corresponding to the oscillation mode of -SH at 2557 cm^−1^, expected to be present in the MPA structure, was absent in the FTIR spectra of the QDs samples. The relatively weaker peak at 2200 cm^−1^ was also attributed to thiol groups. These findings suggested that thiol groups (-SH) reacted with the surface of QDs. The bands anticipated around 2950 - 2957 cm^−1^ in the MPA structure, corresponding to C-H interactions like CH2 and CH3 vibrations, were absent in CdSe/ZnS-MPA samples. The absence of these peaks indicated that no free MPA was released without binding to the surface in the samples. In the QDs spectrum shown in Fig. 4a, peaks at 1462 cm^−1^ and 1630 cm^−1^ were associated with CH2 and NH groups, respectively. The peak observed may be linked to the bending vibration of N–H groups in PEI at this position. Moreover, the peak around 1100 cm^−1^ corresponded to CH_2_ oscillation. The relatively weaker peak at 2200 cm^−1^ was due to thiol groups. The additional peaks found in the FTIR spectrum may be due to impurities in the QDs and other chemical components used in the MPA coating process. According to literature, peaks originating from C-N vibrations were visible around 1402 cm^−1^ and 1503^−1^ cm in the FTIR spectrum of ZnS. The disappearance of these peaks after chemical binding with SPION suggested that the binding may have occurred from these groups [37]. The strong bindings observed could affect delocalized vibrational modes, resulting in significant modifications in frequency and amplitude. The broad and strong peaks at 3300 cm^−1^ related to the hydroxyl (-OH) group indicated the presence of water in the FTIR spectrum [38]. Our data indicated that the fabricated SPION - CdSe/ZnS - aptamer nanocomposite structures were water-soluble and suitable for biosensor applications [2].

The formation of the QDs-SPION aptamer biosensor was also confirmed by UV-vis spectra. PEI-coated SPIONs showed a distinct absorption peak at around 220 nm (Fig. 3). The aptamer showed a broad band in the UV region (300 - 600 nm) [39]. After functionalization of the nanobiosensor with aptamer, this band showed a slight increase in absorbance (Fig. 3), confirming the presence of all components.

### Fluorescence-Based Detection and Quantification of Troponin I

Fluorescence analysis was employed to detect the binding of Troponin I to the QDs-SPION-Aptamer nanobiosensor. For this purpose, fluorescence measurements were conducted using Troponin I concentrations of 2, 5, 10, and 40 ng/mL sequentially. The measurements were performed within the fluorescent emission wavelength range of 500–620 nm. It was observed that the sensor, excited by UV wavelengths, reached its maximum emission point in the wavelength range of 545–550 nm due to the CdSe/ZnS QDs.

As demonstrated in Fig. 4, adding Troponin I increased fluorescence intensity in the sensors after the recognition of troponin by its aptamer. The increased fluorescence of the nanobiosensor in the presence of troponin may be related to the metal-enhanced fluorescence (MEF) via the fluorescence energy transfer (FRET) mechanism [40]. The energy transfer is proportional to the distance (1/d6); therefore, after the binding of the target protein to the aptamer, the intensification of the energy transfer, probably due to the decrease in the distance between the SPIONs and QDs rather than the aptamer, caused the system to increase fluorescence emission. It has been shown in the literature that circular dichroism (CD) studies of the aptamer in the presence of different concentrations of troponin did not cause any conformational changes in the aptamer [41]. This suggests that the distance between SPIONs and QDs may be shortened by troponin with two α-helix structures.

Fluorescence intensity changes showed a linear relationship with increasing Troponin I concentrations, resulting in a strong response. To determine the amount of troponin-dependent fluorescence increase, linear regression analysis was performed. In the (F1-F0)/F0 value, which expresses the relative fluorescence change, F1 represents the fluorescence value generated by the sensor in the presence of troponin, while F0 represents the absence of troponin. An R^2^ value close to 1 indicates that one variable directly affects the other. We observed a linear amplification in the fluorescence signal concentration in the range of 2 - 40 ng/ml troponin. The regression equation is (F1-F0)/F0 (Relative fluorescence intensity) = 0.0156 × Troponin (ng/ml) + 0.2137 (R2 = 0.9777).

The standard deviation method was used to calculate LOD. In particular, the LOD was determined as the product of the threefold standard deviation of the blank absorbance (*n=*3) and the slope of the regression line: LOD=(3.SD)/m, where SD is the standard deviation of the mean blank signal and m is the slope of the linear regression curve. The detection limit of CdSe/ZnS-SPION-Aptamer sensor method was found 13.3 ng/ml and limit of quantification (LOQ) was 40.3 ng/ml.

### Determination of selectivity and stability of fluorescence biosensor

**Fig. 5:**
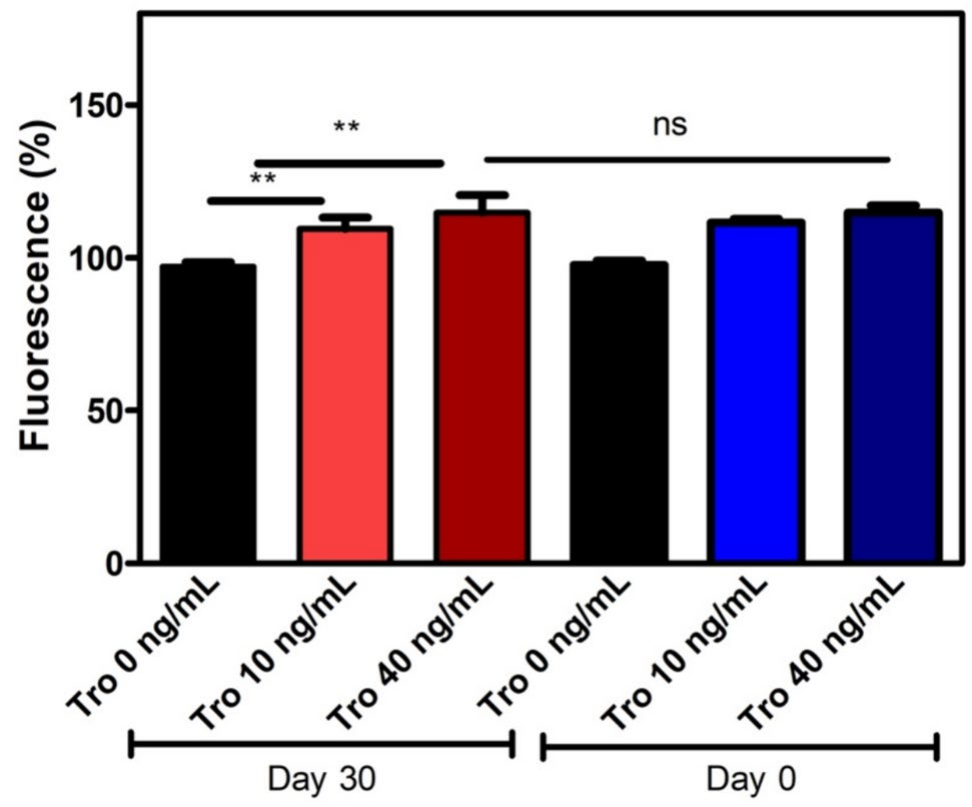
Storage stability and reusability of CdSe/ZnS—SPION—Aptamer sensor within 30 days (∗ *p* < 0.05), ns = not significant

The different concentrations of troponin I were detected with CdSe/ZnS-SPION-Aptamer sensor (Fig. 4) then sensor components were magnetically separated and washed for further usage. The feasibility and the stability of this biosensor for different concentrations of troponin (10 ng/ml and 40 ng/ml) were tested on day 30. We observed the same response as the previous results (p < 0.005) after four weeks when stored in the refrigerator at + 4 ^0^C (Fig. 5). After three reuse cycles, there was no noticeable loss in fluorescence intensity. Our results demonstrated the very high photostability and reusability of CdSe/ZnS-SPION-Aptamer sensor within 30 days. When compared to the stability of sensors from hours to two weeks, our sensor is very useful in terms of its long-term stability [42].

**Fig. 6:**
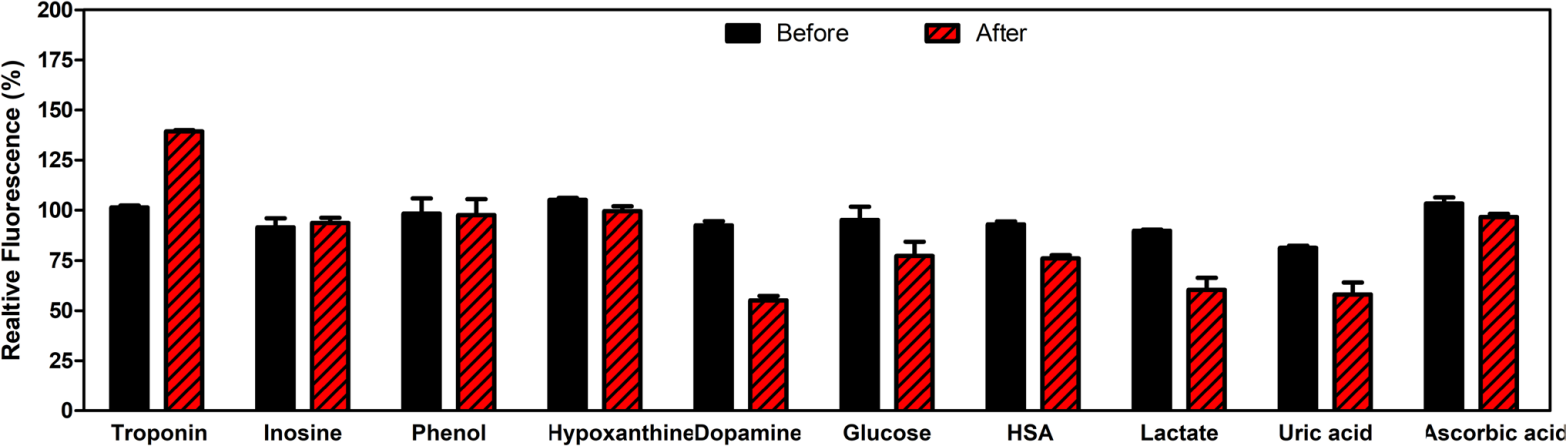
The effect of different molecules and proteins on the fluorescence intensity of the CdSe/ZnS-SPION-Aptamer sensor

To test the selectivity performance of the CdSe/ZnS-SPION-Aptamer sensor, fluorescence measurements were carried out in the presence of some of the chemicals and biomolecules commonly found in human blood serum and cells, such as uric acid, lactate, glucose, ascorbic acid, inosine, dopamine, HSA analytes (0.5 μg/mL). These different analytes were added to the biosensor separately. Then, the fluorescence intensities were analyzed before and after the addition of analytes (Fig. 6). Although the presence of Troponin increased the fluorescence intensity through the “turn-on” mechanism, the fluorescence intensity was significantly quenched when acidic analytes were bound to the sensor. This shows that the normal switch-on mechanism was turned into a switch-off mechanism. pH significantly alters the optical properties of QDs, as both the structure of the QD and the surface binder may be altered depending on pH (Sup. 2). Although QD-based systems generally exhibit fluorescence stability at pH 7–12, protonation of functional groups in acidic environments can cause them to detach from the QD surface, leading to fluorescence suppression [43]. According to the literature, the fluorescence of Cd QDs coated with MPA has been shown to decrease by approximately 20% when exposed to serum albumin [44]. In the presence of Q-Dots and glucose, gluconic acid and -OH radicals may be formed, resulting in fluorescence quenching [45]. Interestingly, the fluorescence intensity of the sensor was half of the initial intensity in the presence of dopamine. It is known that fluorescence quenching of quantum dots has been frequently observed due to the oxidation product of dopamine, benzoquinone. Charge transfer between benzoquinone and proximal groups, such as the MPA cap, could contribute to the fluorescence quenching process [46]. When multiple target analytes need to be determined in a real sample, chromatography is preferable as target analytes can be separated and identified using an analytical column. According to our results, the developed turn-on-fluorescence nanosensor can be used instead of fluorescence quencher as an accurate and sensitive strategy for detecting Troponin I at trace concentrations.

## Conclusions

In the present study, the fabrication of a turn-on biosensor by hybridization of QDs with SPIONs and Tro- 4 aptamer via EDC/NHS chemistry was reported. This ready-to-use fluorescence-based biosensor was used as a sensing platform for Troponin I without the need for pre-treatment or complex instrumentation and can be used repeatedly. Fluorescence measurements in the presence of Troponin I resulted in a significant increase in the fluorescence value even at very low concentrations of troponin. The analytical curve of the CdSe/ZnS-SPION-Aptamer sensor showed good linearity for troponin I concentrations in the range of 2 to 40 ng/ml. The fluorescence increase, rather than decrease, in the presence of interfering metabolites in serum provides acceptable sensitivity, and the satisfactory reusability of the nanosensor showed that the CdSe/ZnS-SPION-Aptamer sensor is a good candidate for troponin I detection.

In future work, this method has the potential to be used in combination with smartphones for on-site detection needs.

## Supplementary Information

Below is the link to the electronic supplementary material.

Supplementary file1 (DOCX 102 KB)

## Data Availability

No datasets were generated or analysed during the current study.
