# Supplementary material for: Reusable Turn-on Fluorescent Biosensor for Cardiac Biomarker Troponin I Detection Using QDs-SPION-Aptamer
Source: J Fluoresc. 2025 Apr 21;35(10):10233–44. doi: 10.1007/s10895-025-04303-0 (PMC12672779; doi:10.1007/s10895-025-04303-0)
Supplement: Supplementary file 1 — Supplementary file1 (DOCX 102 KB) [file 10895_2025_4303_MOESM1_ESM.docx]

**Reusable Turn-on Fluorescence Biosensor for Cardiac Biomarker Troponin I Detection Using QDs-SPION-Aptamer**

**Ayse Ozdemir^1^*, Yigitcan Algül^2^, Nimet Yildirim Tirgil^3^**

*^1^ Ankara Bilim University, Software Engineering, Çankaya, Ankara, Turkey 6570*

*^2^ Ankara Hacı Bayram Veli University, Polatlı Science and Literature Faculty, Departmant of Biology, Ankara, Turkey 06800*

*^3^ Ankara Yıldırım Beyazıt University, Materials Engineering, Faculty of Engineering and Natural Sciences, Biomedical Engineering, Yenimahalle, Ankara, Turkey 06560*

**Supplementary material**

**
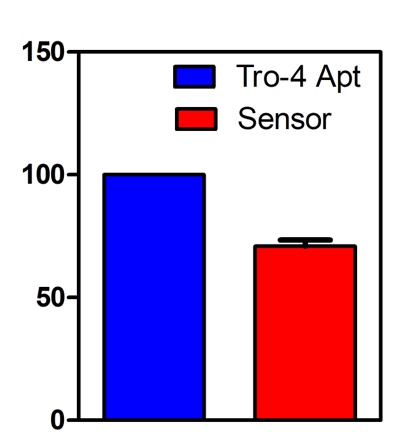
**

Figure 1: The aptamer coating efficiency for the functionalization of the QDs-SPION-Aptamer nanosensor via developed method.

**
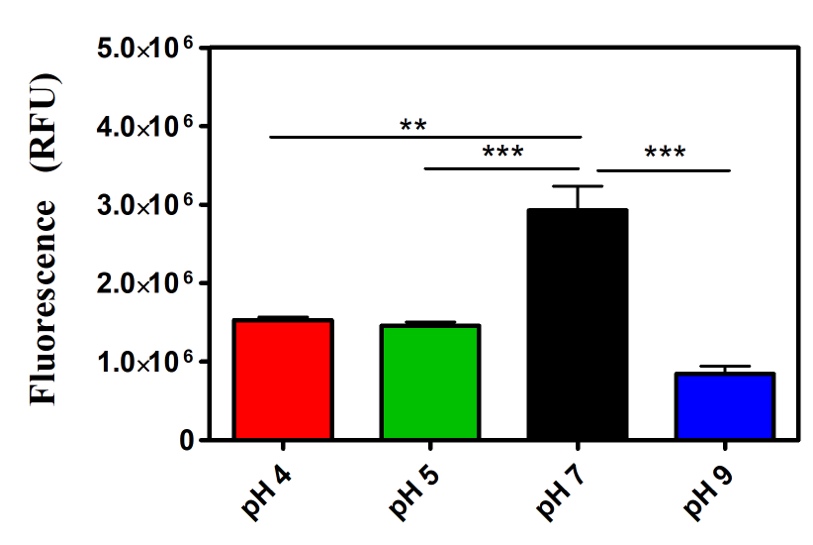
**

Figure 2: The effect of pH on the fluorescence intensity of the CdSe/ZnS QDs.
